# Spatio-temporal variation and socio-demographic characters of malaria in Chimoio municipality, Mozambique

**DOI:** 10.1186/s12936-016-1371-x

**Published:** 2016-06-21

**Authors:** João Luís Ferrão, Jorge M. Mendes, Marco Painho, Sara Z. João

**Affiliations:** Engineering Faculty, Mozambique Catholic University, Chimoio, Mozambique; NOVA Information Management School, Universidade Nova de Lisboa, Lisbon, Portugal; Manica Provincial Directorate of Health, Chimoio, Mozambique

**Keywords:** Malaria, Spatiality, Temporality, Socio-Demographic, Chimoio, Precision health

## Abstract

**Background:**

In Africa, urban malaria is a major concern, since the towns and especially their suburbs are growing quickly. In Mozambique, malaria represents 45 % of all cases of outpatient visits and 56 % of inpatient visits at paediatric clinics. Malaria is a major public health burden in Chimoio Mozambique and few studies on malaria exist.

**Methods:**

The study was carried out to establish the spatiality and temporality of malaria and describe socio-demographic characteristics of malaria patients in Chimoio. Weekly malaria data for 9 years (2006–2014) were collected from the district Epidemiological Bulletin and incidence by season, age, gender, and residence was calculated. SPSS version 20 was used for statistical analysis and ArcGis 10.1 was used to produce maps.

**Results:**

The annual overall average of malaria incidence was 20.1 % and the attributable fraction (AF) of malaria was 16 %. There were differences in weekly and yearly malaria occurrences throughout the period. There was no difference in malaria cases between male and female patients. Children under 5 years of age are three times more prone to malaria than adults (p < 0.05). Three temporal clusters of malaria were identified: cluster 1, weeks 25–47 with average weekly cases of 618 (sd = 251.9), cluster 2, weeks 18–24 and 48–51 with average weekly cases of 1066 (sd = 317.4). cluster 3, weeks 1–17 and 52 with average weekly cases of 1587 (sd = 722.4). Similarly, three different clusters were identified according to residential areas: cluster 1 (10 %) mostly urban, cluster 2 (22 %) mostly suburbs, cluster 3 (28 %) mostly rural areas.

**Conclusion:**

Malaria is increasing in the suburbs, and rural areas present more cases of malaria compared to urban areas. This article is an initial step to understand the dynamics of malaria in Chimoio. Results suggest that malaria varies in time and space, and that precision public health strategy should be used to control malaria occurrence. Studies on weather factors affecting malaria cases, bed net usage, and others should be undertaken.

## Background

Malaria is a very old disease and is a major public health problem in Africa. An estimated 91 % of deaths in 2010 were in the African Region. Most deaths occur amongst children living in Africa, where a child dies every minute from malaria [1, 2]. Urban malaria in Africa is a problem of substantial and growing proportions since these areas are growing quickly, especially in suburbs with poor houses and drainage, farming activities, large amount of vegetation, fruit trees, and persistent poverty. Children and pregnant women are severely and disproportionately affected by malaria in high malaria burden countries [3]. The disease undermines people’s health and capacity to work, hampering the social and economic development of the countries involved [4].

In Mozambique, malaria represents 45 % of all cases in outpatient visits, 56 % of inpatient visits at paediatric clinics, and around 26 % of all hospital deaths [5]. The high prevalence in many parts of the country puts the entire population at risk and poses a challenge for malaria elimination efforts nationally. The peak of malaria occurs during and after the rainy season. Transmission intensity varies from region to region, with high prevalence in areas where climatic conditions are favourable to its development and transmission, whereas some drier parts of the country are epidemic-prone [6].

An association between malaria prevalence and socioeconomic status of households was established in Mozambique. The prevalence of malaria is 43 and 58 % in urban and rural areas, respectively. Pregnant women with high levels of education tend to be more protected against malaria (59 %) compared to non-educated (36 %). Children from better-off families tend to be more protected against malaria (58 %) compared to children from poor families (43 %) [7].

There are conflicting reports regarding the impact of urbanization on malaria endemic trends. Some authors [8, 9] claim that the urbanization process results in profound socio-economic and landscape changes that reduce malaria in urban areas, while some report an increase in malaria in urban regions due to population increase, over-crowding, and poor sanitation [10, 11]. Few spatial studies of malaria have been reported for Mozambique and most studies on malaria variation are based on monthly data [6, 12]. The maps that exist on malaria were produced at the National or Continental level, such as MARA [13], and have limited operational use to support local programme activities.

The patterns of malaria transmission at the local level, especially in Chimoio, have not been studied or precisely defined. This type of research is needed in order to develop cluster risk maps and identify locations and populations at risk for appropriate planning and implementation of targeted and epidemiologically sound preventive and control measures against the disease.

Precision Health is defined as improving the ability to prevent disease, promote health, and reduce health disparities in populations by: (1) applying emerging methods and technologies for measuring disease, pathogens, exposures, behaviours, and susceptibility in populations; and (2) developing policies and targeted public health programmes to improve health [14]. This technique is based on specific site observation, and measuring and responding to variability in disease trends. If related to socio-demographic characteristics, using weekly data, it can lead to decision support systems that help to eradicate disease, optimise resources, and minimise the impact on the environment.

Chimoio is a municipality in the central region of Mozambique. The major cause of death in the municipality in 2013 was malaria 15 % [15]. Swaziland is a neighbouring country of Mozambique with landscape and weather similar to Chimoio Municipality. Using an integrated strategy for malaria eradication achieved a 98 % decrease in reported malaria cases between 2000 and 2011 and seeks to eliminate malaria by 2015 [16].

The goal of this study was to determine the spatial and temporal patterns or clusters of malaria distribution and socio-demographic characteristics of malaria patients in Chimoio Municipality to help decision-making in Precision Public Health strategies in malaria prevention and eradication, using weekly data.

## Methods

### Study area and population

**Fig. 1:**
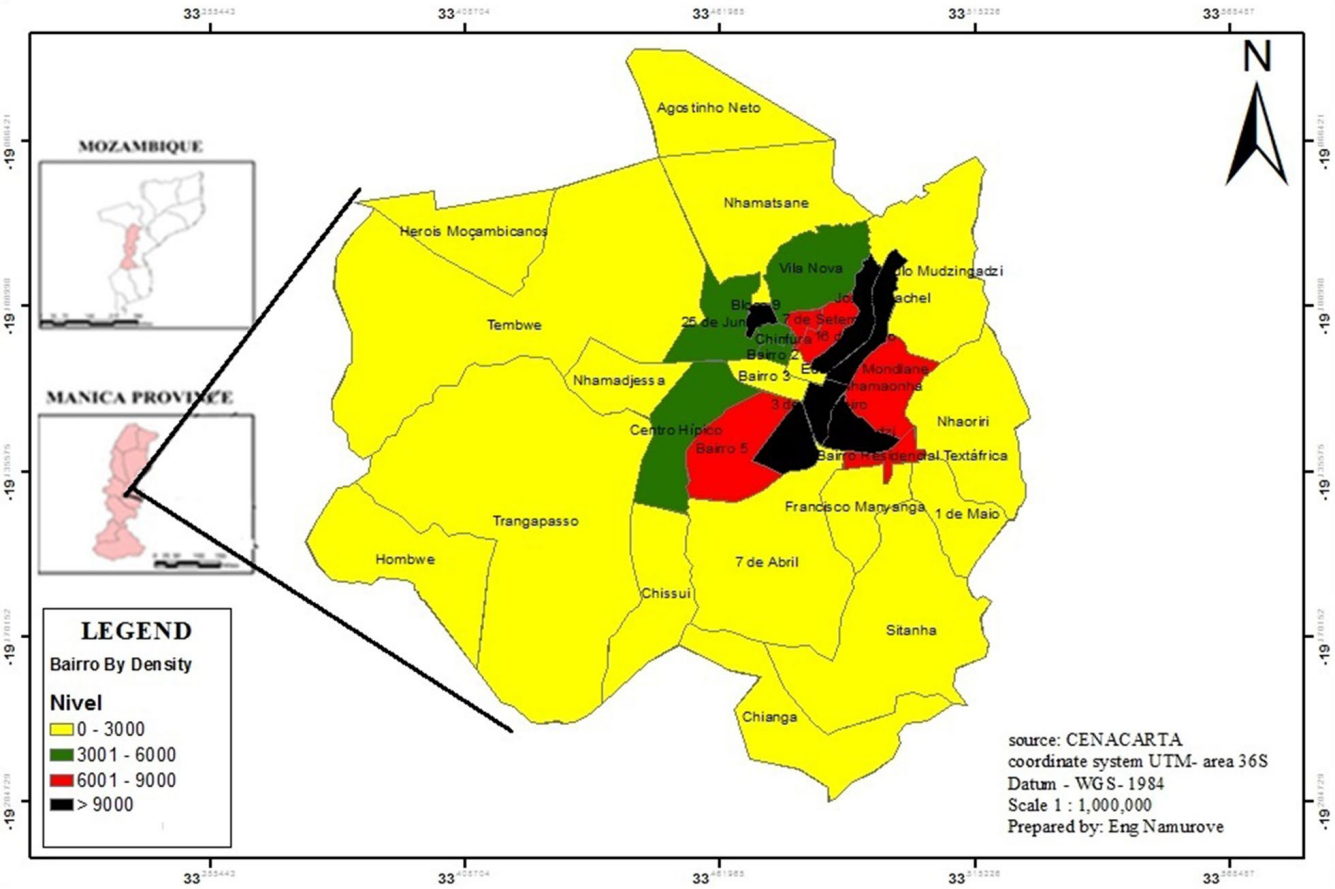
Administrative map of Chimoio, with population density (person/km^2^) Adapted from CENACARTA

**Fig. 2:**
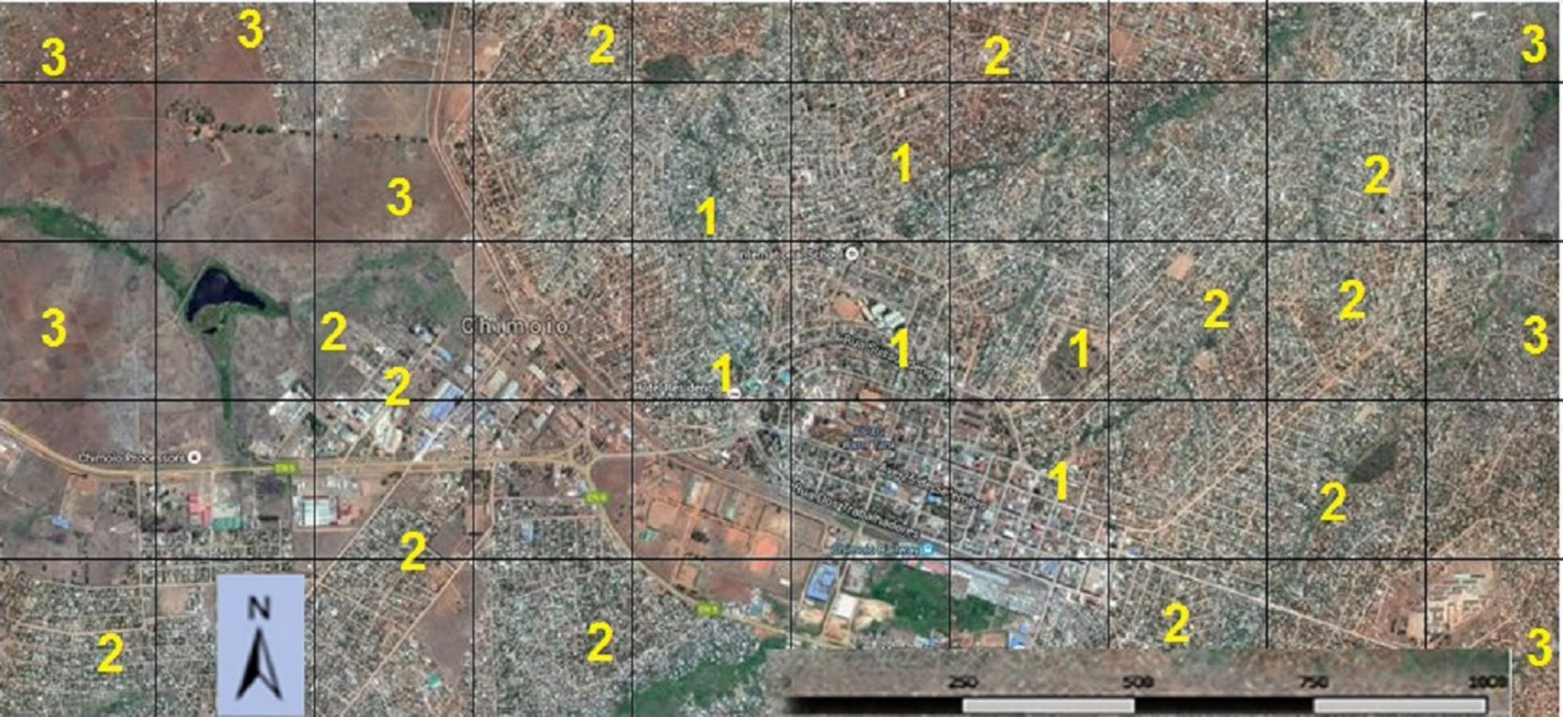
Partial view of Chimoio. 1 = Urban, 2 = Suburb, 3 = Rural Google maps, 20/4/2016

Chimoio is a municipality of Manica Province in the central region of Mozambique, −19°6′59S, 33°28′59E (Fig. 1). The population of Chimoio was 237,497 inhabitants in 2007, with a 3.5 % annual growth rate. Men and women represent 52 and 48 % of the population respectively. Population from 0 to 5 years comprises 17 % of the inhabitants [17]. The city is administratively divided into three urban districts with 33 residential areas called *Bairros*, (Fig. 2) comprised of urban, suburban, and rural areas. The urban area consists of colonial buildings; one to four storey brick houses with large streets and large areas of private open space. There are sewage and sanitation facilities in place. The residents of these areas have medium to high-income levels. On the contrary, most of the suburbs are crowded; some housing units are made of bamboo and wooden poles, and few are of bricks and concrete. In those areas there is poor sanitation, narrow or non-existent streets, and the income levels are low to medium. Rural areas consist of scattered houses, covered with grass, and that are inhabited by low-income residents. There is no electricity and running water and they are continuously expanding.

The area is 174 km^2^ at an altitude of 750 m. Chimoio has a warm temperate climate with dry winters from April to July, hot and dry summer from August to October, and hot humid summer from November to March. The average temperature in Chimoio is 21.5 °C. With an annual rainfall average of 1143 mm, Chimoio has 201 dry, 41 intermediate, and 123 wet days, and the wet period is from 26 November to 29 March [18].

Chimoio has six public health centres’, one Provincial hospital, and two private clinics. The oldest health centers (more than 10 years) are Centro de Saude Eduardo Mondlane (CSEM), Centro de Saude 1^o^ de Maio (CS1Maio), Centro de Saude Namahonha (CSNh) and Centro de Saude Chissui (CSCh). The other two centres, Centro de Saude 7 de Abril (CS7Abril) and Centro de Saude Vila Nova (CSVN) are more recent (less than 5 years).

### Study subjects

In the public health centres and in the provincial hospital malaria cases and other occurrences are compiled daily to produce the Weekly Epidemiological Bulletin (BES) and then sent to the Chimoio Directorate of Health, where data are summarized into a Weekly District Epidemiological Bulletin and channelled to the Provincial Directorate of Health (DPS). Weekly malaria data from the 9 years period (2006–2014) were collected from the district BES: missing bulletins were completed at DPS. The data collected provides information on cases of malaria, gender, and age of the patients. Total malaria cases for each week for the 9 year period (2006–2014) were added and averaged. The aggregated values starting with Week 1 to Week 52 for the 9-year period represent the variables of the study.

Data from the area of residence of the patients were collected from Centro de Saúde Eduardo Mondlane (CSEM), Centro de Saúde 1^o^ De Maio (CS1Maio), and Centro de Saúde Nhamahona (CSNh), (see Map1), which are the largest and oldest clinics in Chimoio Municipality. Proportional randomized sample data from daily record books of the clinics (n = 35,864) were used of which 18,292 were from CSEM, 9185 from CS1Maio, and 8387 from CSNh. Data collected were from 2009 to 2014 due to data availability and aggregated on a weekly basis.

Malaria cases from 2006 to 2009 included cases confirmed either by microscopy or rapid diagnostic test (RDT), and also clinical malaria (fever) diagnosed by health personnel. From 2010 to 2014 malaria cases that were recorded were only from microscopy and RDT. An adjustment for clinical malaria was made for the years 2006–2009 since from 2010 to 2014 of the total fever cases, 78 % were malaria cases.

To compute the attributable fraction (AF) of malaria, monthly data from CSEM were used from 2006 to 2014 and aggregated on a monthly basis. This clinic is the oldest health centre in Chimoio accounts for 57 % of all malaria cases in the period (227,814).

The AF of malaria was calculated using the following formula:

$$AF\;\left( \% \right)\; = \;\frac{Malaria\; Cases}{Total \;Visits}\; \times \;100.$$

Population estimates for Chimoio by locality, gender, and age for the years 2006–2014 were calculated based on the 2007 national census using the mean annual population growth rate for Chimoio of 3.5 %. Average population for the study period was calculated (Table 1).

**Table 1:** Weekly cases of malaria in Chimoio 2006–2014

Year	2006	2007	2008	2009	2010	2011	2012	2013	2014	Overall
Nr. Cases	45,458	60,306	58,333	39,874	41,925	47,107	52,463	60,381	84,707	490,555
Week average	874	1160	1122	767	806	906	1009	1161	1629	1048
SD	316.6	477.4	584.4	397.5	349.4	477.4	498.2	715.4	1126.6	642.1
Max	1751	3059	3086	1697	1783	2170	2257	3217	4438	2606
Min	438	222	397	276	317	332	425	281	489	353
Population	231,462	238,768	250,088	259,764	269,818	280,263	291,116	302,392	314,108	270,864^a^
Year incidence (%)	19.6	25.3	23.3	15.4	15.5	16.8	18.0	20.0	27.0	20.1^b^

^a^Average Population

^b^average cases/divided by average population × 100

### Data analysis

Total malaria cases per week were derived from the collected data adjusted to the population increase per year (3.5 %). The malaria incidence per 100 person-year was calculated from the total number of cases occurring in each week in each Bairro divided by the total person-week and then multiplied by 100.

$$Incidence \;rate \left( \% \right)\; = \;\frac{No.\;of\;cases}{Population \;Size}\; \times \;100.$$

Linear trend analysis, and multi-way ANOVA to test difference between years and weeks using Tukeys’ test for mean separation were performed. Chi square for proportion of gender and age and Phi, Cramer’s V test was used for statistical significance. Regression analysis to test association between malaria cases and population density of residential areas was performed. Temporal and spatial hierarchical cluster analysis using square Euclidean distance was performed to identify cluster between the weeks and between residential areas in malaria incidence and dendrograms were produced. All tests were performed using Microsoft Excel (Analysis Tool pak) and SPSS IBM version 20; spatial maps were produced to analyse spatial variation along the years using ArcGis version 10.1.

## Results

### Malaria cases and incidence in Chimoio

Table 1 reports that malaria cases in Chimoio between 2006 and 2014 amounted to 490,555. In 2010 the fewest cases were recorded, 41,925, and then in 2014 they almost doubled to 84,707. The incidence of malaria decreased from 2006 to 2010 and increased from 2012 to 2014. The annual average incidence of malaria was 20.5 % with a maximum of 25.3 % in 2007 and a minimum of 15.5 % in 2010.

**Fig. 3:**
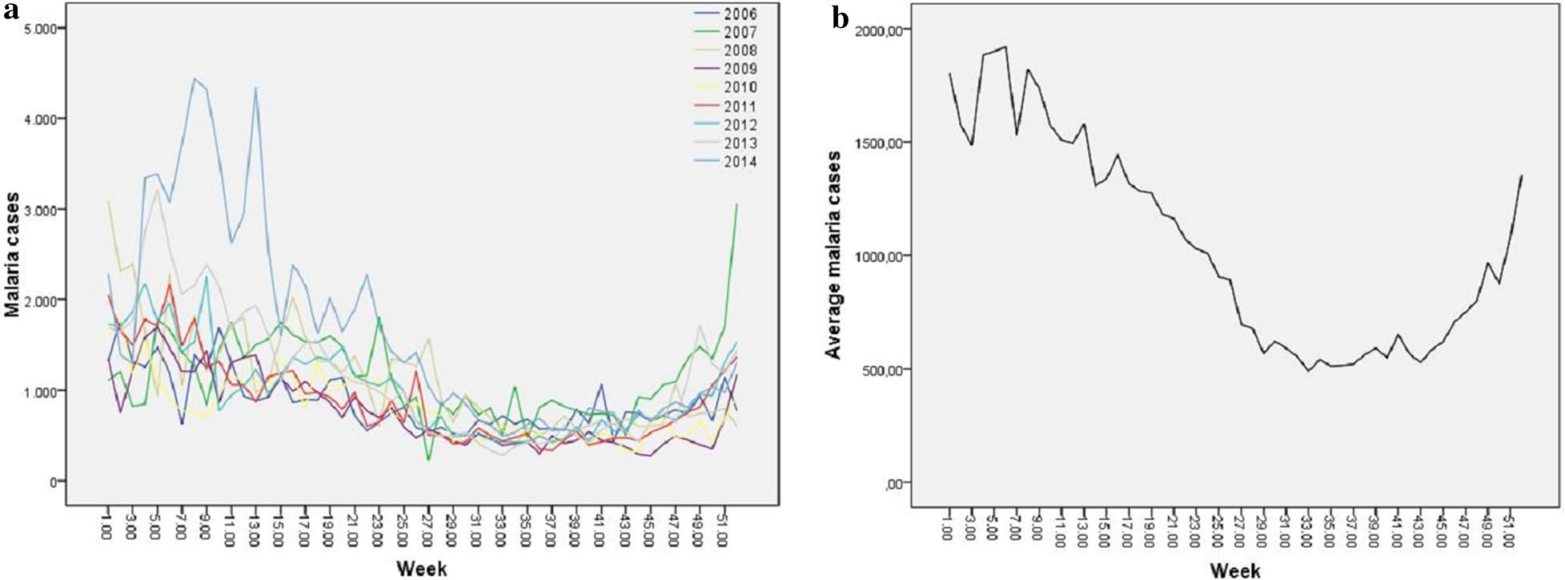
Weekly malaria Cases in Chimoio 2006 to 2014. Annual (**a**), Average (**b**)

Figure 3 presents the temporal linear trend of malaria in Chimoio. Figure 3a shows the temporal distribution of Malaria for each year and Fig. 3b shows the average variation over the 9 years. In terms of weeks, the lowest week recorded was 222 cases per week in week 27 in 2007 and the highest record was 4438 in week 8 in 2014. There is a high weekly variation in malaria cases ranging from 222 to 4438 cases per week, CV, 61 % (1A).

On average week 6 presents the highest cases of malaria with 1919 cases and week 33 the lowest 419 (3B). Malaria weekly average was 1048 (sd 642.1) cases ranging from 806 cases in 2010 to 1629 cases in 2014. Malaria cases (1048) presented a difference between years (p < 0.05), the calculated F value was 22.1 while the critical table value was 1.96. Malaria cases (1048) also shows a difference between weeks (p < 0.05), the calculated F value was 11.55 while the critical table value was 1.38.

Table 2 presents the summary and the mean separation of malaria cases between the years. Years 2009 and 2010 differs from years 2006, 2007, 2008, 2011, 2012, and 2013; and year 2014 differs from the other years in malaria cases.

**Table 2:** Summary and malaria cases mean separation between years

(years)	2009	2010	2006	2011	2012	2008	2007	2013	2014
*N*	52	52	52	52	52	52	52	52	52
Mean*	767^a^	806^a^	874^b^	906^b^	1009^b^	1122^b^	1160^b^	1161^b^	1629^c^
*SD*	397.5	349.43	316.56	477.44	498.16	584.45	477.45	715.42	1126.62

^a, b, c^ Different letters indicate difference between years. Tukeys’ test (p < 0.05)

### Temporal clusters of malaria in Chimoio

**Fig. 4:**
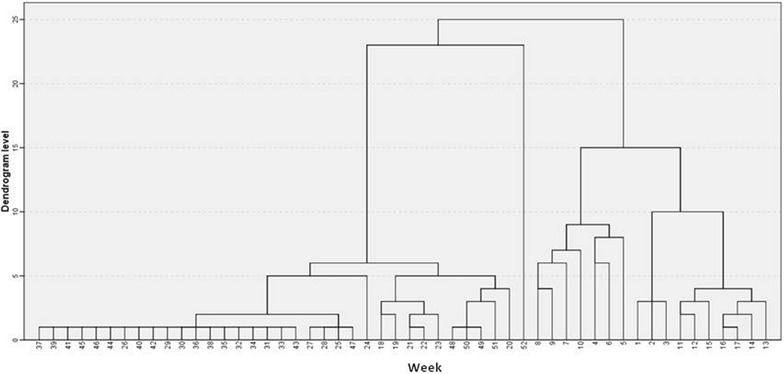
Temporal Cluster of Malaria in Chimoio 2006–2014

A temporal cluster analysis of malaria occurrence was performed and the results are summarized in the dendrogram in Fig. 4. Three temporal clusters of malaria were identified: cluster 1 comprises weeks 25–47 with an average of weekly cases of 618 (sd = 251.9), 44 % of the total weeks, representing the dry season; cluster 2 comprises weeks 18–24 and 48–51 with an average for weekly cases of 1066 (sd = 317.4), 21 % of the total weeks, the intermediate season; Cluster 3 comprises weeks 1–17 and 52 with an average for weekly cases of 1587 (sd = 722.4), 35 % of the total weeks, the wet season.

There is a difference (p < 0.05) between cluster 1 (618), cluster 2 (1066) and cluster 3 (1587). The calculated F value was 185.35 while the critical table value was 3.02. Table 3 presents the malaria cases mean separation between the three clusters.

**Table 3:** Summary week cluster malaria mean separation

*Groups*	Cluster 1	Cluster 2	Cluster 3
*N*	207	99	162
Mean*	618^a^	1066^b^	1587^c^
*SD*	215.89	371.37	722.42

^a, b, c^ Different letters indicate difference between years. Tukeys’ test (p < 0.05)

#### Malaria cases by gender and age

In terms of gender, there is no overall difference (p > 0.05) between women (51 %), and men (49 %), between adult women (52 %) and adult men (48 %), or female children (50.3 %) and male children (40.7 %), Chi square 2, df = 1. In terms of age, out of 490,555 cases of malaria in Chimoio, 235,830 (48 %) were in children under 5 years and 254,725 (52 %) in patients over 5 years old. A difference (p < 0.001) is clear between these two groups, the calculated Pearson Chi Square was 48; df = 1.

### Attributable fraction of malaria in Chimoio

**Fig. 5:**
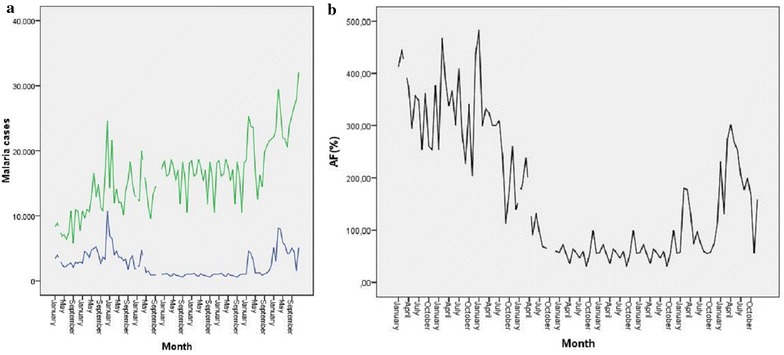
**a** Malaria cases and patient visits. **b** Attributable fraction of Malaria

Figure 5 presents the trend of malaria cases and patient visits to CSEM from 2006 to 2014 and the monthly AF. From 2006 to 2014 CSEM was visited by 1885,195 patients and 259,252 were tested positive for malaria (5A). The monthly peak of malaria was in February for years 2006, 2007, 2010, 2011, 2012, 2013, March for years 2009 and 2014, and December for year 2008 (5B). The annual average AF of malaria was 16 %.

Years 2010, 2011, and 2012 recorded the lowest AF of malaria in Chimoio, 5.7 % and the year 2009 the highest, 27 %. There is no difference (p > 0.05) in AF malaria among years, the calculated Pearson Chi Square was 54.00, df = 48. There is a difference (p < 0.001) in AF malaria among months, the calculated Pearson Chi Square was 913.349, df = 693.

### Relationship between malaria cases and population density

Figure 1 presents the Chimoio population density per bairro, and the population density varies from 28 to 17,049 (person/km^2^). A regression analysis was performed to determine the association between population density by residential area and malaria cases. From Table 4 it is clear that there is a medium positive correlation, r = 0.407 between malaria cases and population density and the r^2^ value indicates 0.165, which implies that 16.5 % of malaria cases are attributed to population density. At 0.05 significance level the calculated F value is 5.741 while the critical table value is 1.344. Thus, malaria cases significantly depend on population density.

**Table 4:** Model summary regression malaria cases and population density

Model	R	R square	Adjusted R square	Std. error of the estimate	Change statistics					Durbin–watson
					R square change	F change	Df1	Df2	Sig. F change	
1	0.407^a^	0.165	0.136	1529.737	0.165	5.741	1	29	0.023	1.617

^a^Predictors: (constant), population density (km^2^)

^b^Dependent variable: Malaria Cases

Furthermore, from the coefficients in Table 5, the beta value is positive (0.407), That is, as population density increases malaria cases increase as well.

**Table 5:** Regression coefficients

Model	Unstandardized coefficients		Standardized coefficients	T	Sig.			Collinearity statistics	
	B	Std. error	Beta			Lower bound	Upper bound	Tolerance	VIF
(Constant) Population density (Km^2^)	1071.398	400.498		2.675	0.012	252.289	1890.508		
	0.145	0.061	0.407	2.396	0.023	0.021	0.269	1.000	1.000

Malaria cases and population density

Dependent variable: malaria cases

#### Malaria clusters per residential area

**Fig. 6:**
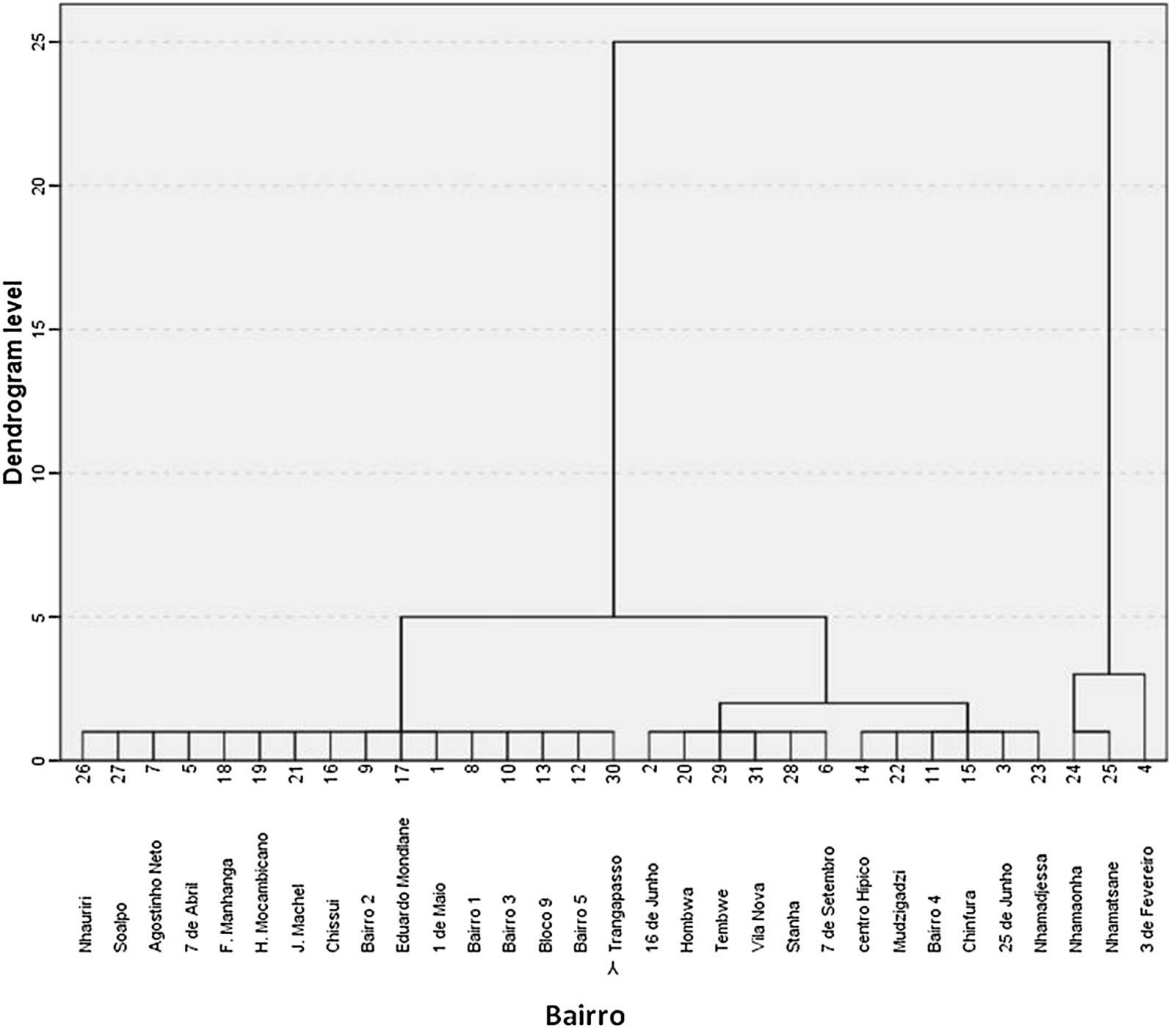
Malaria clusters by residence in Chimoio 2006/2014

Figure 6 presents the dendrogram clusters by residential areas. Three clusters were identified. Cluster 1 comprises the following bairros: Bairro 1, 2, 4, Herois Mocambicanos, 7 de Abril, 7 de Setembro, Bloco 9, Nhamatsane, Tembwe, Agostinho Neto and Eduardo Mondlane. The average incidence is 10.6 % and represents 35 % of the Bairros of the municipality, most of them urban areas. Cluster 2, comprises the following Bairros: Hombwa, Josina Machel, 3 de Fevereiro, Vila Nova, Mudzingazi, Bairro 3, 5, Nhamadjessa, Nhamaonha, Francisco Manyanga, 25 de Junho, Centro Hipico, Textafrica, 16 de Junho, and Chinfura. The average incidence is 21.9 % and represents 8 % of the Bairros of the Municipality, most of them are suburbs. Finally, cluster 3 comprises the following Bairros: Nhauriri, Chissui, Sitanha, 1 de Maio and Trangapasso. The average incidence is 28.4 % and represents 16 % of the bairros of the municipality, which are mostly rural areas. There is a difference (p < 0.05) between malaria clusters, and the calculated Pearson Chi Square was 7.99.00, df = 2.

### Malaria prevalence from 2010 to 2014

**Fig. 7:**
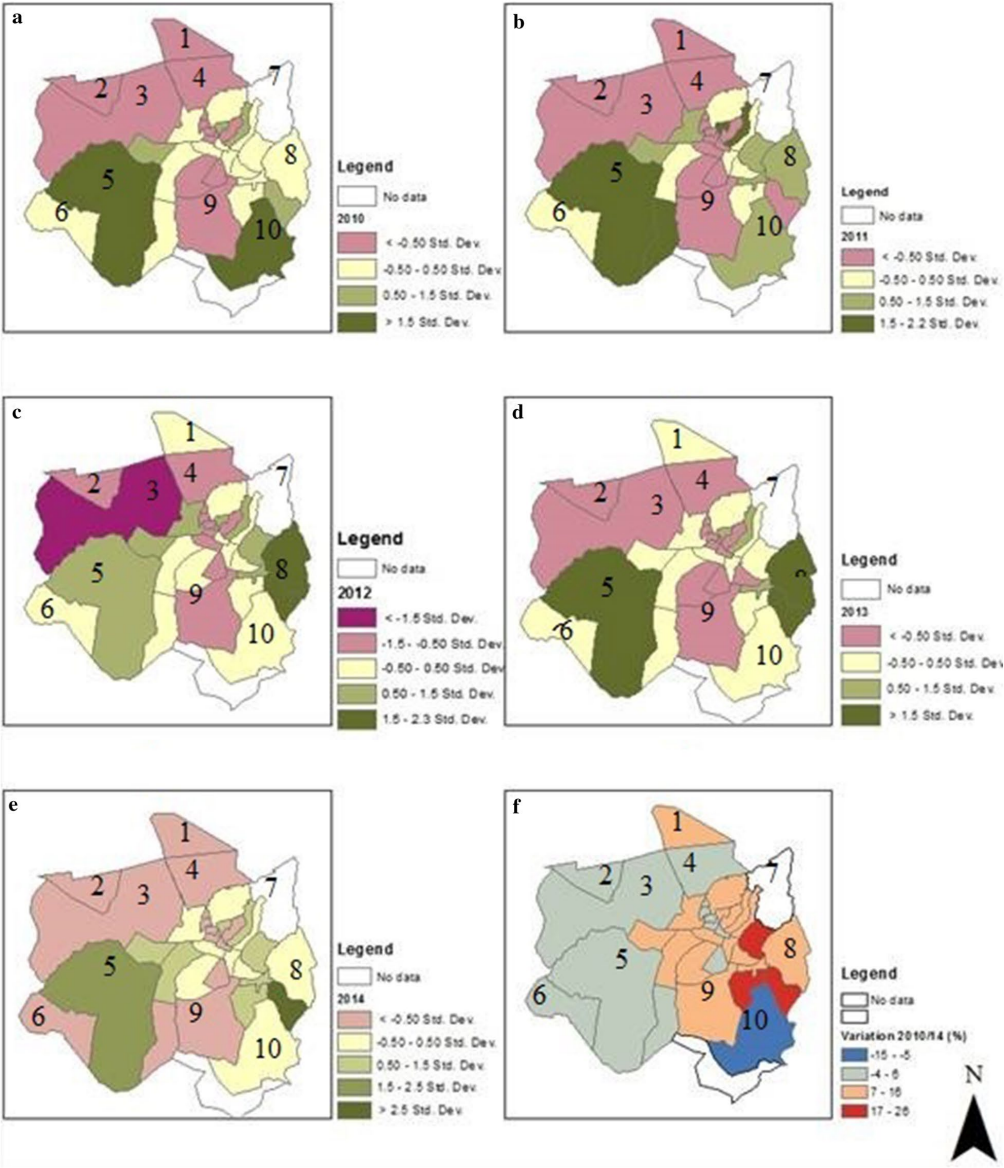
Incidence of malaria. **a**–**e** Spatial distribution. **f** Spatial variation. Chimoio Bairros: 1 = Agostinho Neto, 2 = Herois Moçambicanos, 3 = Tembwe, 4 = Nhamatsane, 5 = Trangapasso 6 = Hombwa, 7 = Circulo Mudzingadzi, 8 = Textafrica, 9 = 7 de Abril, 10 = Stanha

Figure 7 depicts the incidence of malaria for the 5 years under analysis (2010–2014). Figure 7a–e shows the spatial distribution of malaria for each year and Fig. 7f shows the spatial variation over the 5 years. It is possible to see that the incidence of malaria varies spatially in the Chimoio Municipality. The areas located in the north of the municipality (Agostinho Neto, Herois Mocambicanos, Tembwe, and Hombwa) show values consistently below average of up to 1.5 standard deviations whereas areas located in the southeast (Trangapasso) and southwest (Nhaurire and Textafrica) of the district show values consistently above the average of up to 2.5 standard deviations.

In terms of malaria variation, from 2009 to 2014 Sitanha, the most rural bairro, presented a reduction of malaria incidence from 5 to 15 %; Nhamatsane, Herois Mocambicanos, Tembwe, and Hombwa, rural bairros, and Bairro 1, 2, 3, 4, and Bloco 9 urban bairros, presented a reduction of 4–6 %. Bairros 1^o^ de Maio, Francisco Manhanga and Nhamaonha, new suburbs, presented the highest increase of 7–26 % and the rest of the bairros, which are old suburbs had an increase from 7 to 16 %. Overall, and for the 5 years, malaria incidence has increased between 7 and 26 % in the bairros situated in the central eastern part of the municipality (suburbs) and decreased between 4 and 15 % in the other areas (rural and urban).

## Discussion

Malaria epidemiology has never been investigated in the study area before. The overall annual incidence of malaria in Chimoio was 20.5 %. Maputo city reported 15.7 % [19] and Manica Province 43 % [7]. Incidence of malaria in Chimoio is lower than in Manica Province due to the fact that Chimoio is an urban area and residents have more resources for malaria prevention, and the weather is cooler than in many parts of the country. Overall malaria cases and incidence have been increasing in Chimoio in recent years, especially in suburbs. After a decrease in 2010 with 41,925 cases reported and 15.5 % incidence, 84,704 cases were reported in 2014, showing an incidence of 27 %.

These results are in concordance with a study of patterns and trends of malaria conducted in Kenya that found an increase of 111.13 and 109.52 % per annum in 1988–2002 and 1998–2005 respectively for morbidity and hospitalization [20]. This increase in the Chimoio results is probably due to the 3.5 % annual increase in population, reduced efforts to combat malaria and persistent poverty. However this is contrary to reports of a substantial reduction of malaria incidence in sub-Saharan Africa [2, 21].

The greatest number of cases occurs mostly in February (peak of the rainy season) and the fewest number of cases in September (dry season). This is in concordance with [6] who finds that the peak of malaria in Mozambique occurs during the rainy season. It should the noted that the 20.5 % incidence may be overestimated as it does not consider the same people being diagnosed more than once in a year, or underestimated since generally there are cases that are not reported, especially those which are far from health centres, self medication, and use of traditional healers.

It was reported that in Chimoio the land cover is changing towards less vegetation [8, 9, 22, 23, 24] claim that the urbanization process results in profound socio-economic and landscape changes that reduce malaria in urban areas, this is in line with findings of this study that the incidence of malaria is decreasing in rural areas. The results of this study differ from those in Amazonia, where the population increase resulted in occupation of more space and increases in disease incidence [11, 25]. There is a difference in malaria occurrence between years and this is in line with [1, 6].

Contrary to most research [3, 21], this study did not find any difference between women and men in malaria cases, between adult women and adult men, or between female children and male children. The chances of getting malaria are the same (gender equality) in Chimoio. This can be explained by the high malaria incidence in the area, which puts the entire population at risk [6].

There was a difference between children under 5, (48 %) and population over five (52 %). Children under five comprise 17 % of the population of Chimoio but accounted for 48 % of the malaria cases, almost three times more. This disproportion is also reported by other authors [3, 7]. This is probably due to the fact that children under 5 years of age have little immunity to the malaria parasite.

Very few studies have been carried out using weekly data [26, 27]. Most use monthly data and differentiate malaria cases between dry and rainy season or cooler and hot seasons [28–30]. The results of this study almost coincide with the results of Westerink [18] who reported that Chimoio has 201 dry (29 weeks), 41 intermediate (6 weeks), and 123 wet days (17 weeks), suggesting that malaria cases vary in these three periods. In terms of Precision Public Health, the three distinct periods should have different approaches regarding prevention and combat.

The attributable fraction of malaria was 16 %. In Mozambique 45 % of all cases in outpatient visits are due to malaria [3] and in Manica Province 43 % [7], meaning that the AF of malaria in Chimoio is almost a third of the province and the country value. This may be due to the cooler weather and the fact that country averages include rural areas, where there is more malaria than in cities, and in the cities there are more malaria control interventions.

In terms of malaria cases related to population density, there was a medium positive correlation r = 0.407 between the population density and malaria incidence and the R^2^ value indicates 0.165, which implies that 16.5 % of malaria cases are attributed to population density. The extremes of both low and high population density modify malaria transmission and have profound consequences for estimates of Chimoio´s Mozambique public health burden.

In terms of residential areas, annual malaria incidence varies from 9 to 45 %, meaning that Chimoio is an area with hypo endemic and mesoendemic malaria [31]. In terms of malaria spatiality, in rural areas malaria incidence is decreasing, probably due to the reduction in vegetation cover and deforestation, and in urban areas probably due to availability of better measures and living conditions. In suburban areas malaria incidence is increasing, probably due to increasing poverty, poor sanitation and poor living conditions. Other studies report the same pattern of findings [7, 21].

## Conclusions

This study concludes that in Chimoio the incidence of malaria presents a spatial and temporal pattern. Malaria cases have been increasing over the years, especially in suburbs, and there is a difference in malaria cases by year and weeks. There is no gender difference in malaria cases. Children under 5 years of age are three times more prone to get malaria than the rest of the population. There are three different periods of malaria in Chimoio: hot and rainy season, dry and cool season, and hot and dry season (dry, wet, and intermediate). Sixteen percent of visits to the health centres are from malaria patients. The rural areas of the municipality have more malaria cases, followed by suburbs, and urban areas have fewer malaria cases. Overall, and for the 5 years studied malaria incidence has increased between 7 and 26 % in the bairros situated in the east central part of the municipality (suburbs) and decreased between 4 and 15 % in the other areas (rural and urban).

Precision Public Health strategies that target malaria weekly according to the positive cases, and temporal and spatial distribution can be formulated to combat and eradicate malaria in Chimoio Municipality. Studies on weather and climate factors affecting malaria, bed net usage, and others should be undertaken.
